# Irinotecan pharmacokinetics-pharmacodynamics: the clinical relevance of prolonged exposure to SN-38

**DOI:** 10.1038/sj.bjc.6600447

**Published:** 2002-07-02

**Authors:** R H J Mathijssen, J Verweij, W J Loos, P de Bruijn, K Nooter, A Sparreboom

**Affiliations:** Department of Medical Oncology, Erasmus MC–Daniel den Hoed, PO Box 5201, 3008 AE Rotterdam, The Netherlands

**Keywords:** irinotecan, SN-38, pharmacokinetics, pharmacodynamics, limited sampling

## Abstract

We have shown previously that the terminal disposition half-life of SN-38, the active metabolite of irinotecan, is much longer than earlier thought. Currently, it is not known whether this prolonged exposure has any relevance toward SN-38-induced toxicity. Here, we found that SN-38 concentrations present in human plasma for up to 3 weeks after a single irinotecan infusion induce significant cytotoxicity *in vitro*. Using pharmacokinetic data from 26 patients, with sampling up to 500 h, relationships were evaluated between systemic exposure (AUC) to SN-38 and the per cent decrease in absolute neutrophil count (ANC) at nadir, or by taking the entire time course of ANC into account (AOC). The time course of SN-38 concentrations (AUC_500 h_) was significantly related to this AOC (*P*<0.001). Based on these findings, a new limited-sampling model was developed for SN-38 AUC_500 h_ using only two timed samples: AUC_500 h_=(6.588×C_2.5 h_)+(146.4×C_49.5 h_)+15.53, where C_2.5 h_ and C_49.5 h_ are plasma concentrations at 2.5 and 49.5 h after start of infusion, respectively. The use of this limited-sampling model may open up historic databases to retrospectively obtain information about SN-38-induced toxicity in patients treated with irinotecan.

*British Journal of Cancer* (2002) **87**, 144–150. doi:10.1038/sj.bjc.6600447 www.bjcancer.com

© 2002 Cancer Research UK

## 

Irinotecan (CPT-11) belongs to the family of camptothecins, and is a member of the class of topoisomerase I inhibitors. A broad spectrum of anti-tumour activity was seen in preclinical models as well as in patients, with responses observed in various disease types, including colorectal, lung, cervical, and ovarian cancers (Mathijssen *et al*, 2001; Vanhoefer *et al*, 2001). CPT-11 is a prodrug that requires activation to the active metabolite, 7-ethyl-10-hydroxycamptothecin (SN-38), which is approximately 100- to 1000-fold more active than the parent drug (Hertzberg *et al*, 1989). A host of enzymes, including carboxylesterases to form SN-38 (Humerickhouse *et al*, 2000), UDP glucuronosyltransferases mediating SN-38 glucuronidation to form the β-glucuronic acid conjugate, SN-38G, (Iyer *et al*, 1998), intestinal and endogenous β-glucuronidases causing deconjugation of SN-38G (Sparreboom *et al*, 1998; Slatter *et al*, 2000), as well as cytochrome P-450 isoforms (Haaz *et al*, 1999; Santos *et al*, 2000) to form APC and NPC are involved in CPT-11 metabolism. In addition, several drug-transporting proteins, notably a canalicular multispecific organic anion transporter (cMOAT) located on the bile canalicular membrane (Chu *et al*, 1998), and P-glycoprotein (Chu *et al*, 1999), can influence CPT-11 elimination through hepatobiliary secretion and intestinal (re-)absorption.

As a result of these (and probably also other presently unknown) mechanisms, CPT-11 and its metabolites are subject to large interindividual kinetic variability (Mathijssen *et al*, 2002a), and show relatively long disposition half-lives. We have recently shown that by applying an extended sampling-time period of 500 h, the circulation time of SN-38 in cancer patients was found to be substantially longer than held previously (Kehrer *et al*, 2000). We hypothesised that, because of the poorly defined relationships between pharmacokinetic parameters and pharmacodynamic outcome of CPT-11 treatment (Mathijssen *et al*, 2001), the prolonged terminal-disposition phase of SN-38 should be taken into consideration in future studies to identify kinetic correlates that would assist in prediction of the dose-limiting myelosuppression. Here, we examined the impact of this prolonged SN-38 exposure using an *in vitro* approach with a panel of human cancer cell lines, and re-examined exposure-toxicity relationships in a group of 26 cancer patients treated with CPT-11 administered as a 90-min intravenous infusion in a 3-weekly regimen at a dose of 350 mg m^−2^.

## MATERIALS AND METHODS

### Growth-inhibition studies

**Figure 1:**
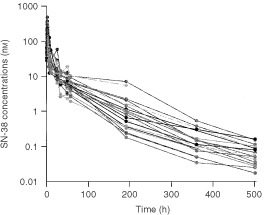
Plasma concentration-time profiles of SN-38 in patients treated with a 90-min i.v. infusion of CPT-11 (dose level 350 mg m^−2^).

Exponentially growing cells obtained from the IGROV (ovarian cancer), Caco-2 (colon cancer) and H226 (lung cancer) cell lines were cultured in 4 ml of RPMI-1640 medium, and plated in 25 cm^2^ T-flasks (Corning Costar), at a density of 10 000 cells/flask. Hereafter, plates were incubated in a controlled environment at 37°C and 95% air per 5% CO_2_, and 24 h after the start of incubation, the medium was replaced by medium containing SN-38 at final concentrations of 100 pM (39.2 pg ml^−1^), 1.00 nM (392 pg ml^−1^), or 10.0 nM (3920 pg ml^−1^). These concentrations were chosen because they adequately simulated the median SN-38 plasma concentrations circulating in patients after 3 weeks, 10 days and 2 days, respectively, after infusion of the standard dose of CPT-11 (350 mg m^−2^, given as a 90-min infusion once every 3 weeks; see Figure 1

). As a control, flasks containing cells in the absence of SN-38 were used. At 48-h intervals, and for a maximum period of about 3 weeks (i.e., 500 h), the culture medium was replaced with fresh medium to minimise possibly confounding effects caused by instability of SN-38 through chemical instability. At the same intervals, culture flasks were treated with trypsin for 30 min, and subsequently the cell suspension was centrifuged for 2 min at 500 **g** (4°C). Next, the supernatant was discarded, and the cells were re-suspended in 50 μl of buffer and counted in a Bürker-cell chamber. Each experiment was repeated twice, with each counting performed in quadruplicate.

### Patients and treatment

We retrospectively studied records from patients participating to various prospective clinical trials on CPT-11 in which pharmacokinetic monitoring was involved; the full clinical profiles are documented elsewhere (Kehrer *et al*, 2000, 2001a, 2002). All patients had a histologically or cytologically confirmed malignant solid tumour for which CPT-11 was considered to be a treatment option. An adequate haematopoietic (white blood cell count (WBC) ⩾3.0×10^9^ l^−1^; absolute neutrophil count (ANC) ⩾1.5×10^9^ l^−1^; and platelet count (PLT) ⩾100×10^9^ l^−1^), hepatic and renal function at the time of study entry was required. Eligibility criteria also included a World Health Organization performance status of 0 or 1, and age between 18 and 70 years. Before study entry, written informed consent was obtained from all patients.

CPT-11 was provided by Aventis (Antony Cedex, France) as a hydrochloride trihydrate salt in vials containing 40 or 100 mg, dissolved in d-sorbitol and a lactic acid-sodium hydroxide buffer system (pH 3.5–4.5) at a concentration of 20 mg ml^−1^. This solution was further diluted in 250 ml of sterile, isotonic sodium chloride, prior to dosing. The drug was administered as a 90-min continuous intravenous infusion at a dose of 350 mg m^−2^, and each patient received prophylactic antiemetics including ondansetron (8 mg) or granisetron (1 mg) and dexamethasone (10 mg). In the first course of treatment, patients did not receive any other medication known to interfere with CPT-11 pharmacokinetics.

### Pharmacokinetic analysis

Blood samples for pharmacokinetic analysis were drawn from an indwelling cannula from the arm opposite to that used for drug infusion. These samples were collected in glass tubes containing lithium heparin prior to infusion, at 0.5, and 1.5 h (end of infusion) during infusion, and at 10, 20, and 30 min, and 1, 1.5, 2, 4, 5, 8.5, 24, 32, 48, 56, 190, 360, and 500 h after the end of infusion. Blood samples were immediately centrifuged at 3000 **g** for 10 min after collection to separate the blood cells and the plasma fraction, and were stored at −80°C until analysis.

Concentrations of CPT-11 and its metabolite SN-38 were determined by reversed-phase high-performance liquid chromatography with fluorescence detection, as described in detail elsewhere (De Bruijn *et al*, 1997). To determine SN-38 at low femtomole levels, a more specific analytical method was used (De Bruijn *et al*, 1999). Earlier pharmacokinetic findings showed that total drug levels (i.e., the total of lactone and carboxylate forms) of CPT-11 and SN-38 provided a consistent and accurate reflection of the pharmacologically active lactone concentration of both compounds with little variability (Sasaki *et al*, 1995). Therefore, it was concluded that total drug monitoring of CPT-11 and SN-38 could serve as an appropriate surrogate of the respective lactone forms. For practical reasons, we only used total drug forms for further analysis. Plasma concentration-time data of CPT-11 and SN-38 were fitted to a tri-exponential equation using the software package Siphar v4.0 (InnaPhase, Philadelphia, PA, USA), based on previously described considerations for model discrimination (Sparreboom *et al*, 1998). Calculation of the area under the plasma concentration-time curve (AUC) was based on integration from the tri-exponential curve with extrapolation to infinity. Extrapolated AUC values of SN-38 were either based on a 56-h (AUC_56 h_) or a 500-hour sampling-time period (AUC_500 h_).

### Exposure-toxicity relationships

**Figure 2:**
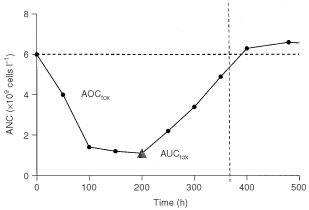
Example of an ANC-time pattern. The triangle represents the nadir count. The horizontal line represents the baseline value of ANC.

Haematological parameters of interest (i.e., those derived from ANC, WBC and PLT) were determined before CPT-11 infusion and on a twice weekly basis during the entire pharmacokinetic sampling-time period, to enable a pharmacokinetic/pharmacodynamic (PK/PD) evaluation. The pharmacodynamic endpoint for establishment of the PK/PD relationships was determined in two different ways. (i) First, prior to the PK/PD analysis, individual toxicity data expressed as blood cell counts were plotted for each patient in a concentration *vs* time profile. The estimated area over these curves (AOC_tox_), as well as the area under the curves (AUC_tox_), was calculated by the linear-trapezoid method (Figure 2

). The ratio AOC_tox_/(AOC_tox_+AUC_tox_), referred to as AOC, in which the denominator represents the area described by the pre-therapy toxicity value times the maximum pharmacokinetic sampling time, was hypothesised to be a simple and accurate function of drug-related haematological toxicity (Gurney *et al*, 1998). (ii) Second, drug-related haematological toxicities were evaluated by using the nadir count, which was measured to determine the per cent decrease in haematopoietic cells as:


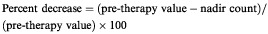


To avoid confounding bias due to changes in haematological data unrelated to treatment, patients showing extreme pre-infusion haematological numbers (i.e., with ANC>13.0×10^9^/liter^−1^) were excluded in these calculations. Each of these two relative haematological-toxicity functions were fitted to the AUC of SN-38 using a sigmoidal maximum effect (E_max_) model, based on a modified Hill equation, as follows:


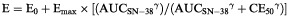


In this equation, E_0_ is the minimum reduction possible, E_max_ is the maximum response, CE_50_ is the AUC value of SN-38 predicted to result in 50% of the maximum response, and γ is the Hill constant, which describes the sigmoidicity of the curve (Holford and Sheiner, 1981).

Pharmacokinetic-pharmacodynamic analysis was performed using the software package Siphar v4.0, and weighted and extended least squares techniques were used to estimate model parameters. Model selection was guided by the decrease in the objective function value (−2×log likelihood). The criteria to assess the goodness of fit of the models was based on: (i) computation of the coefficient of variation, defined as the ratio of the standard deviation computed using the variance-covariance matrix and the parameter value, (ii) the Akaike information criterion, and (iii) the correlation coefficient and *P*-value of the relationship.

### Statistical considerations

All statistical calculations were performed using the Number Cruncher Statistical System package v5.X (J.L. Hintze, East Kaysville, UT, USA) and/or the Siphar package. The level of significance was set at *P*<0.05.

### Limited sampling models (LSMs)

Patients were randomly entered in two groups, a training-data set and a validation-data set containing up to 20 samples for each individual patient, as has been described before (Mathijssen *et al*, 1999). A restricted block randomisation was performed as described elsewhere (Altman, 1991) to keep the number of patients close for both groups, and only one course of each patient was taken to avoid bias as a result of potential cumulative effects and to keep the observations independent.

Our goal was to predict the AUC of SN-38 adequately with a minimum number of samples (1 to 3). Moreover, these samples should be taken at the most convenient time-points for practical reasons. The first step in the model development consisted of univariate linear-regression analysis to determine the best single-sample time points from the training data set. Next, we performed a stepwise (forward) multivariate linear-regression analysis using the optimal single sampling-time points to describe the association between SN-38 concentrations at multiple time points and its corresponding AUC. The Pearson's coefficient (*r*), root mean-square error (RMSE), and mean-predictive error (MPE), which describe correlation, precision and bias, respectively, were calculated to determine the best LSMs. The usefulness of the models was evaluated in the training data set, by comparing AUC values determined using the full data with AUC values predicted by the LSMs.

## RESULTS

### *In vitro* analysis of SN-38–induced cytotoxicity

**Figure 3:**
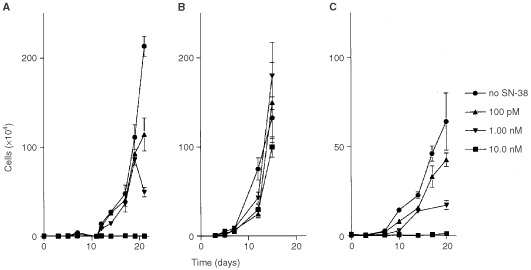
Cell-growth *vs* time curves for the IGROV (**A**), Caco-2 (**B**), and H226 (**C**) cell lines, incubated with medium containing 100 pM, 1.00 nM or 10.0 nM of SN-38 or medium without SN-38 (reference) for a 500-h incubation time period. Data indicate mean values (symbol) with SD (error bars), which are shown when larger than symbol.

The growth-inhibitory potential of low SN-38 concentrations was evaluated *in vitro* against several cell lines using a 3-week continuous-exposure period. Clear manifestation of growth-inhibiting effects were seen at SN-38 concentrations of 100 pM, 1.00 nM and 10.0 nM in the IGROV cell lines, with the number of remaining viable cells being reduced by 46±8.7%, 77±10.4%, and >99.9%, respectively, as compared to untreated controls (Figure 3

). At these concentrations, 34±5.9%, 73±4.0% and 98±0.2% growth inhibition, respectively, was seen in the H226 cells, relative to their controls. As the doubling time of the Caco-2 was shorter than that of the IGROV cells, data could not be obtained for the entire 3-week period in the Caco-2 cell line. Nonetheless, the data suggest overall that the low circulating concentrations of SN-38 determined previously in patients receiving CPT-11 at a dose of 350 mg m^−2^ induce substantial inhibition of cancer cell growth, in spite of evident concerns in extrapolating data from *in vitro* experiments to the clinical situation.

### Patient population

A total of 26 patients, predominantly suffering from colorectal cancer, were entered onto the current trial (Table 1

**Table 1:**
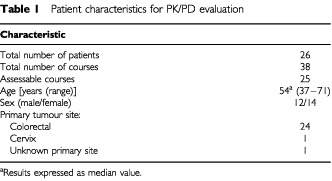
Patient characteristics for PK/PD evaluation

). All patients received a single-agent regimen with CPT-11 in the first treatment course at a dose of 350 mg m^−2^. One course was excluded because of extreme pre-treatment values for haematological parameters (i.e., ANC value greater than 13.0×10^9^ l^−1^), making a total of 25 courses assessable for PK/PD analysis.

### Pharmacokinetics and pharmacodynamics

**Figure 4:**
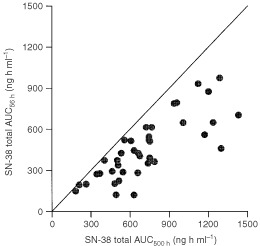
Relationship between calculated SN-38 AUCs, based on 500-h sampling and 56-h sampling respectively. (*n*=38). The solid line represents the line of identity (y=x).

The two groups of SN-38 AUCs based on short (up to 56 h) and prolonged sampling (up to 500 h), respectively, were highly correlated (*r*^2^=0.79). However, as shown in Figure 4

, all AUC values calculated using the short sampling-time period substantially underestimated the true AUC, albeit with huge variation (7 up to 81%). The mean dose over AUC ratio for SN-38 based on prolonged sampling was 860±410 L h^−1^ in this group of patients, and was 1400±890 L h^−1^ when based on short sampling.

**Figure 5:**
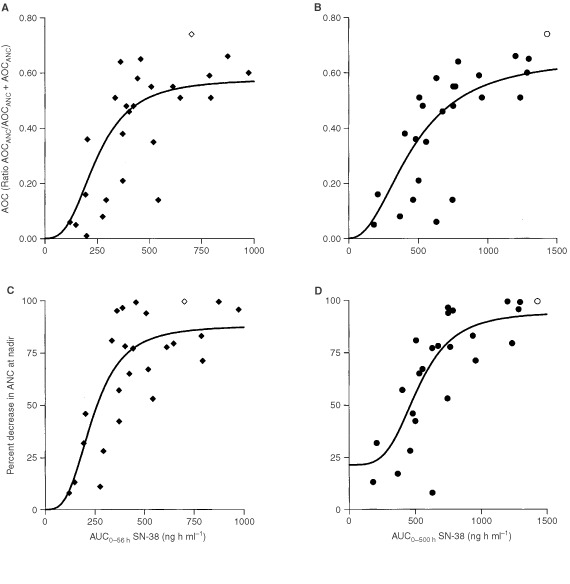
Relationship between ratio AOC_ANC_/(AOC_ANC_+AUC_ANC_) and SN-38 AUC based on a 56-h sampling time period (**A**), or SN-38 AUC based on a 500-h sampling time period (**B**). Relationship between per cent decrease in ANC at nadir *vs* SN-38 AUC based on a 56-h sampling time period (**C**), or SN-38 AUC based on a 500-h sampling time period (**D**). (*n*=25). Open symbols represent excluded data as a result of a baseline ANC>13.0.

The parameter estimates for the different pharmacodynamic models are shown in Figure 5

. Preliminary results demonstrated that separate estimation of the observed maximum effect did improve the fits, and that optimal results were obtained with a model fitted to the data using weighted least squares (weighting factor, 1/y) and the Powell minimization algorithm. Data sets based on short and prolonged sampling were both plotted separately against individual values of ANC, WBC and PLT applying the ratio AOC_tox_/(AOC_tox_+AUC_tox_) and the per cent decrease in blood cell counts, using the E_max_-model (Gianni *et al*, 1995; Ohtsu *et al*, 1995). The kinetic parameters predicted to result in half of the maximum response showed a substantial degree of interindividual variability, and were poorly estimated in some models, presumably because of the small number of patients. The exposure-neutropenia relationship was best described with the model based on the 500-h sampling-time period and the ratio AOC_tox_/(AOC_tox_+AUC_tox_) (Figure 5B), although no significant improvement of the fit was obtained as compared to the relationship based on the short sampling time period (Figure 5A). In the optimal model, the following model parameters were observed: CE_50_=460±82.9 ng h ml^−1^, γ=2.22±1.55, *P*=0.0001 and *r*=0.77. With the use of the per cent decrease in ANC at nadir as pharmacodynamic measure, the fits were worse than those based on AOC. The disparity in the data of ratio AOC_ANC_/(AOC_ANC_+AUC_ANC_) and per cent decrease in ANC suggests that PD analysis based on per cent decrease in ANC is not sufficiently accurate to be used as a measure for prediction of haematological toxicity. In contrast to ANC, no correlations with PK were observed for WBC and PLT.

### Limited-sampling models

We developed a new limited-sampling model (LSM) for determination of SN-38 AUC_500 h_ using one to three samples taken after drug administration, taking into account feasibility and usefulness in a routine clinical setting. There were no significant differences in patient characteristics between the training and validation data set. The same 26 patients were included in this analysis, and in both groups 13 patients were entered (six females and seven males in both cases). The median age in the training set was 53 years (range, 37 to 71 years), *vs* 54 years (range, 42 to 71 years) in the validation set. Results of a univariate-linear regression analysis of SN-38 concentrations at each sample time point and the corresponding AUC showed correlation coefficients ranging from 0.08 to 0.82 in the training set (Table 2

**Table 2:**
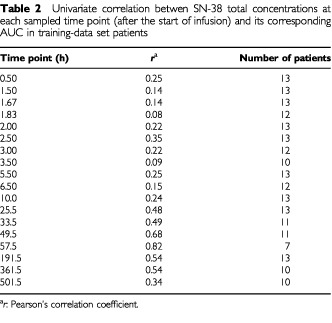
Univariate correlation betwen SN-38 total concentrations at each sampled time point (after the start of infusion) and its corresponding AUC in training-data set patients

). Multiple-regression analysis, based on one or two additional time point led to significantly improved correlation coefficients. Based on a variety of considerations, including low values for RMSE and MPE with high correlation coefficients, the optimal LSM was a bivariate model containing concentration data at two time points


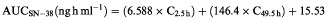


where C_2.5 h_ and C_49.5 h_ represent plasma concentrations of SN-38 (in ng ml^−1^) at 2.5 h after the start of infusion (1 h post-infusion) and 49.5 h (48 h post-infusion), respectively. In both the training set and validation sets, this model showed little bias (MPE, 0.33% and 0.76%) and excellent precision (RMSE, 4.9% and 9.1%), with correlation coefficients of 0.99 and 0.96, respectively (Figure 6

**Figure 6:**
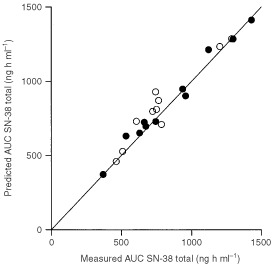
Correlation between the observed AUC from a linear three-compartment model and the predicted AUC from the LSM for SN-38 total. Closed symbols represent training set data; open symbols represent validation set data. The solid line represents the line of identity (y=x).

).

Trivariate models were also evaluated using the clinically most optimal sample-time points (i.e. 1, 190 and 360 h post-infusion). Unfortunately, the predictive performance of this model appeared to be worse (validation set: MPE=1.6%; RMSE=18%; *r*^2^=0.83), despite acceptable training set statistics (MPE=0.62%; RMSE=9.5%; *r*^2^=0.95).

## DISCUSSION

This study shows that the prolonged exposure at low circulating concentrations of the pharmacologically active CPT-11 metabolite, SN-38, in plasma of cancer patients after a single dose has important cytotoxic potential *in vitro* and therewith may have clinical relevance toward SN-38-induced toxicity and antitumour activity. In our *in vitro* experiments we exposed several cancer cell lines to SN-38 concentrations, which were known to be circulating in plasma for up to 3 weeks after a 90-min i.v. infusion of CPT-11 at a dose of 350 mg m^−2^. After a 3-week incubation period in the presence of SN-38 at concentrations of 100 pM, 1.00 nM and 10.0 nM, significantly reduced tumour growth was observed as compared to untreated cells, suggesting significant cytotoxic activity, even at these low drug levels. Using pharmacokinetic data from 26 patients treated with CPT-11, with sampling up to 500 h after drug administration, PK/PD relationships were evaluated between systemic exposure (AUC) to SN-38 and the per cent decrease in ANC at nadir or by taking the entire time course of ANC into account (AOC), using a modified Hill function (sigmoidal E_max_ model). Taking into consideration the entire time course of SN-38 concentrations (AUC_500 h_), the most optimal relationships were observed for the AOCs. These data not only emphasise the need to apply appropriate PK/PD models with sufficient sampling-time points for the accurate estimation of complete concentration-time profiles, but also has direct clinical relevance in view of the fact that relationships between drug exposure and effect (i.e., toxicity and efficacy) were previously poorly defined (Mathijssen *et al*, 2001).

We have shown earlier that the use of an extended sample-collection period of 500 h led to terminal disposition half-life and AUC estimates for SN-38 after CPT-11 administration of 47±7.9 h and 2.0±0.79 μM h, respectively (Kehrer *et al*, 2000), both representing a two-fold increase as compared to earlier reported estimates. As an explanation for this phenomenon, we previously observed a substantial formation of SN-38 from CPT-11 and the cytochrome P-450 3A4-mediated metabolite, NPC, by plasma carboxylesterases. In addition, transport studies in Caco-2 cell monolayers indicated that SN-38 could cross the membrane from the apical to the basolateral side, indicating the potential for re-circulation processes that can prolong circulation times (Kehrer *et al*, 2000). More recently, we also noted extensive partitioning of CPT-11 in erythrocytes resulting in prolonged association of the parent drug in the central blood compartment (Mathijssen *et al*, 2002b). Regardless of the exact underlying causes for the prolonged SN-38 circulation, the fact that low circulating levels of SN-38 demonstrate profound cytotoxic potential indicates the need to not take an oversimplified view when modelling drug-induced haematological toxicity.

Pharmacokinetic information on the concentration-time profiles has previously been combined with efficacy-toxicity data to form PK/PD models for drugs in a wide variety of therapeutic areas. The most common type of this kind of PK/PD model is one in which a summary measure of exposure, such as AUC, is related to the principal side effect of the treatment, in this case haematological toxicity, for which singly measured nadir counts are usually employed. However, experimental evidence suggests that information on an entire time course of changes in blood cell counts is more important than the nadir count. For example, it has been demonstrated that patients with prolonged neutropenia have a greater risk of infection than patients who have the same nadir count with rapid recovery. Therefore, we hypothesised that using data from the entire time course of neutropenia after CPT-11 treatment would provide useful information. Thus, we analysed our data using two different types of analysis (per cent decrease in toxicity at nadir and a measure of time course (AOC)) using two different variables based on AUC calculated using 56-h or 500-h sampling periods, with those based on AOC showing superiority for prediction of haematological toxicity. For both PD parameters, the quantity of available data is positively correlated with the quality (accuracy) of the determined outcome variables. Thus, at least several data points are required for an accurate estimate of the nadir count. In our current study, a rich data set was available with haematological toxicity data, which might explain the moderate predictive performance of the PK/PD relationship using (assumed) nadir counts. Clinically even more important PD parameters than the just mentioned parameters may be (i) the time period during which the neutrophil count remains below a certain level (for instance 4×10^9^ cells l^−1^) or (ii) the area between the curve of time *vs* leukocyte count and the line representing a cell count of 4×10^9^ cells l^−1^ (Minami *et al*, 2001). Also in our current study, this area was shown to demonstrate the best correlation of all tested PK/PD combinations. In order to further optimise PK/PD relationships, a population model for CPT-11-induced neutropenia by modelling the full time course of haematological toxicity is currently being developed, as done previously for paclitaxel and etoposide (Karlsson *et al*, 1998; Minami *et al*, 1998, 2001).

Some clinical implications from the data presented in this study, particularly with respect to considerations of treatment schedule, are easily envisaged. Regarding administration regimens of CPT-11 used in cancer patients, it is particularly noteworthy that different schedules are being used in Europe (350 mg m^−2^ once every 3 weeks), the USA (125 mg m^−2^ weekly for 4 or 6 weeks) and Japan (100 mg m^−2^ every week, Kehrer *et al*, 2001b). Furthermore, there is a current trend for the use of protracted intravenous or oral dosing regimens of CPT-11 using daily drug administration (Kehrer *et al*, 2001b), based on earlier clinical experience with camptothecin analogues with relatively short terminal disposition half-lives, such as topotecan. However, the unique pharmacokinetic behaviour of SN-38, coupled to our current pharmacodynamic observations, suggests that a single administration of CPT-11 repeated every third week is the preferred regimen, and that increased frequency of administration may result in drug accumulation.

Obviously, the clinical applicability of SN-38 AUC as PK parameter predicting toxicity would increase by the availability of accurate and predictive LSMs. Earlier developed models for the determination of SN-38 total AUC were only useful for the estimation of AUC models up to 24 or 56 h (Mathijssen *et al*, 1999). Therefore, a new limited-sampling model (LSM) was developed for determination of SN-38 AUC_500 h_ using only two timed samples: AUC_500 h_ (ng h ml^−1^)=(6.588×C_2.5 h_)+(146.4× C_49.5 h_)+15.53, where C_2.5 h_ and C_49.5 h_ are plasma concentrations at 2.5 and 49.5 h after start of CPT-11 infusion, respectively. The model was shown to be precise (RMSE=4.9%) with no demonstration of any bias (MPE=0.33%). This model enables us to perform PK studies in a very efficient manner, and it is less expensive and might therefore be applicable to large-scale investigations. For example, PK sampling might be performed easily in an outpatient setting. In addition, the use of this LSM may open up historic databases for a re-evaluation, or at least provide prospective studies to obtain information about SN-38-mediated (haematological) toxicity in cancer patients treated with CPT-11. For the determination of SN-38 AUC, this model is currently being implemented in a multi-institutional clinical trial with CPT-11 administered at a dose level of 350 mg m^−2^ in the presence or absence of the aminoglycoside antibiotic, neomycin.

In conclusion, we have shown that the prolonged circulation of SN-38 at low concentrations following CPT-11 administration has clinical implications, and resulted in altered exposure-toxicity relationships. This information, coupled to the prospective implementation of the newly developed LSM, may be of importance for future individualisation of drug therapy in order to avoid excessive toxicity.
